# Intraoperative rapid molecular diagnosis validates MRI‐based glioma boundary evaluation: A case report

**DOI:** 10.1111/cns.14233

**Published:** 2023-04-23

**Authors:** Huizhong Chi, Qingtong Wang, Zhe Han, Xueen Li, Kailiang Zhang, Mei Qi, Zichao Feng, Deze Jia, Bo Han, Shilei Ni, Hao Xue, Gang Li

**Affiliations:** ^1^ Department of Neurosurgery, Qilu Hospital, Cheeloo College of Medicine Shandong University Shandong China; ^2^ Institute of Brain and Brain‐Inspired Science Shandong University Shandong China; ^3^ Shandong Key Laboratory of Brain Function Remodeling Shandong China; ^4^ Department of Pathology Shandong University Qilu Hospital Shandong China; ^5^ Department of Pathology Shandong University School of Basic Medical Sciences Shandong China

Dear Editor,

The relationship between the extent of glioma resection and the actual clinical benefit of patients depends on the balance between cytoreductive surgery and neurological morbidity.^1^ Studies have shown that intraoperative MRI (iMRI) can reduce residual tumor volumes while maintaining a low risk of new neurological deficits.^2^ However, in addition to application safety and economic considerations, image quality can be negatively affected by artifacts from a variety of factors that can lead to biased detection results, including edema, metal sensitivity, radio frequency (RF) noise from improperly shielded electronic devices in the operating room, and brain–air interfaces.^3^, ^4^, ^5^ The fifth edition of the World Health Organization (WHO) Classification of Tumors of the Central Nervous System, published in 2021, proposes new criteria for tumor classification.^6^ Therefore, the analysis of molecular detection has become increasingly indispensable for the need for precise diagnosis and therapy for glioma. However, the detection methods based on immunohistochemistry (IHC) and next‐generation sequencing (NGS) take a long time and are mostly applied to postoperative diagnosis, which cannot meet the needs of intraoperative detection.^7^ The intraoperative rapid molecular diagnosis based on automatic integrated gene detection system (AIGS) real‐time fluorescence PCR is a newly developed technology to analyze the existence of molecular markers relevant to the diagnosis of specific tumor subtypes within 55 min.^8^ We report a case of lower‐grade glioma (LGG) based on the technology, the surgeon verified the accuracy of iMRI in localizing tumor boundaries and achieved precise tumor resection at the imaging and molecular levels (Figure 1A, Video S1).

**FIGURE 1:**
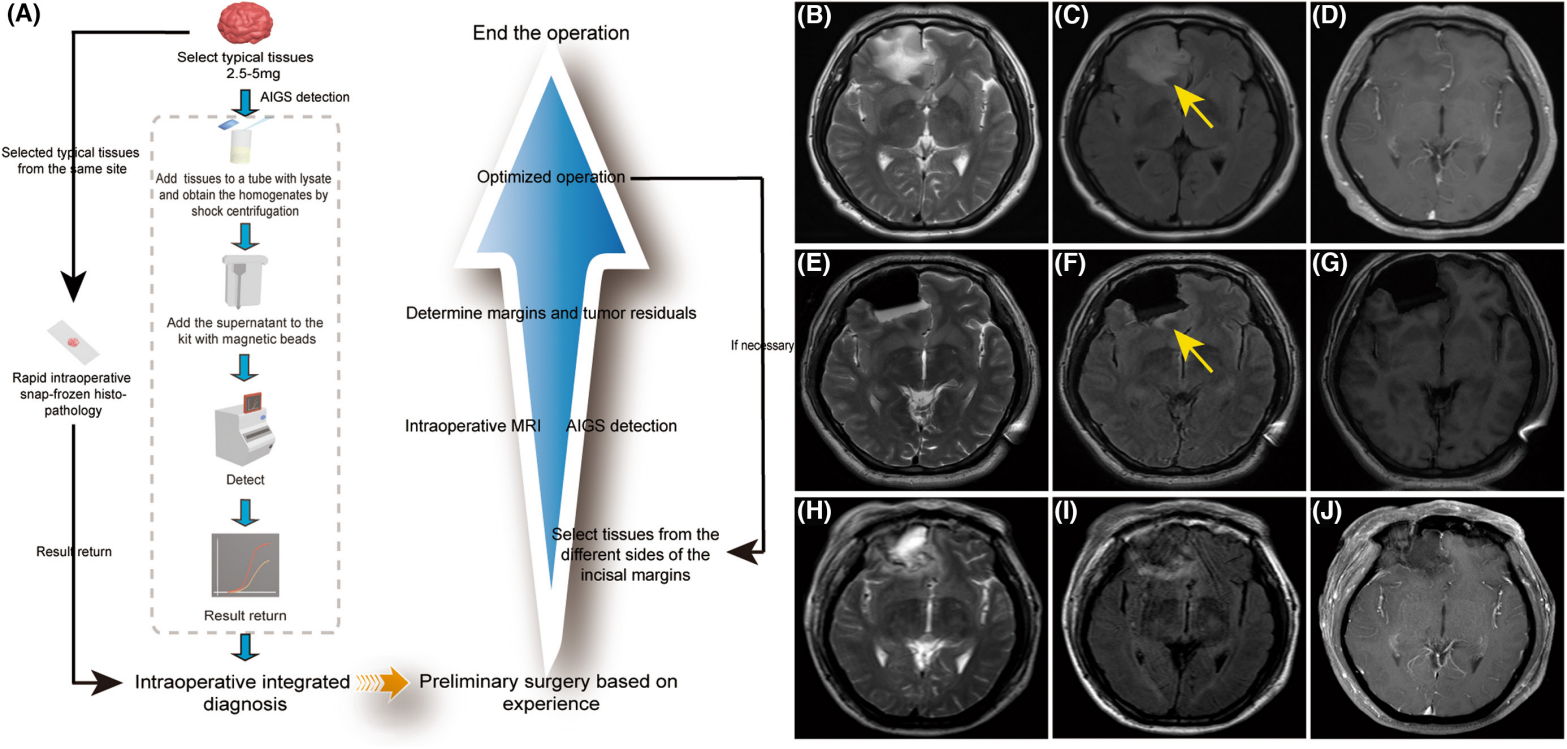
The whole process of the combination of intraoperative rapid molecular diagnosis based on AIGS real‐time fluorescence PCR and intraoperative MRI in glioma resection (A). The preoperative craniocerebral MRI (B–D) of the patient confirmed the location of the lesion. The iMRI (E–G) showed there might be remains of the tumor (yellow arrow) co‐located with the preoperative craniocerebral MRI. The postoperative MRI (H–J) performed 5 days after the surgery showed no obverse lesion signals.

On May 6, 2022, a 48‐year‐old male suffered a sudden loss of consciousness with convulsions and incontinence that lasted about 10 min. A craniocerebral MRI was performed after he was admitted to the hospital. A space‐occupying lesion, possibly a glioma, was discovered in the right frontal lobe of the brain, with T2 and Flair high signals and no significant enhancement, resulting in an epileptic seizure (Figure 1B–D). On May 20, 2022, the patient underwent resection of the space‐occupying lesion in the right frontal lobe. The consent of patient and his family for the procedure was obtained.

We selected typical tissues from the same site to be submitted for intraoperative frozen histopathology and rapid molecular diagnosis, respectively. Resect the lesion along the periphery based on experience. About 30 min later, a low‐grade glioma was considered by rapid frozen histopathology examination. Another 25 min later, the isocitrate dehydrogenase 1 (IDH1) R132H mutation and telomerase reverse transcriptase promoter (TERTp) C250T mutation were indicated by rapid molecular diagnosis (Figure 2A,B). Based on intraoperative frozen histopathology and rapid molecular diagnosis, we inferred that the patient had lower‐grade glioma with the IDH1 R132H mutation and the TERTp C250T mutation. Almost at the same time, the resection of the space‐occupying lesion based on experience was completed. Subsequently, we selected tissues from the anterior, lateral, and posterior sides of the incisal margins for another IDH1 mutation detection. Meanwhile, iMRI was performed for about 30 min. There were some abnormal signals shown by the iMRI co‐located with preoperative MRI lesions, which indicated some remnants of the tumor may exist (Figure 1E–G). Interestingly, the molecular detection selected from the same site also showed the IDH1 mutation still existed at the posterior side, indicating tumor remnants when we finished the iMRI (Figure 2C–E). A further resection was performed. We selected another tissue at the newly formed incisal margin for IDH1 mutation molecular detection, and there were no mutations (Figure 2F). Following that, a craniocerebral MRI 5 days after surgery revealed no significant abnormal signals (Figure 1H–J), indicating complete tumor resection at both the imaging and molecular levels. In addition, the integrated diagnosis based on the conventional histopathological (Figure 2G,H) and NGS results were oligodendroglioma, WHO grade 2, with IDH1 R132H and TERTp C250T mutations, which confirmed the accuracy of intraoperative testing results. The last follow‐up showed no significant neurological deficits.

**FIGURE 2:**
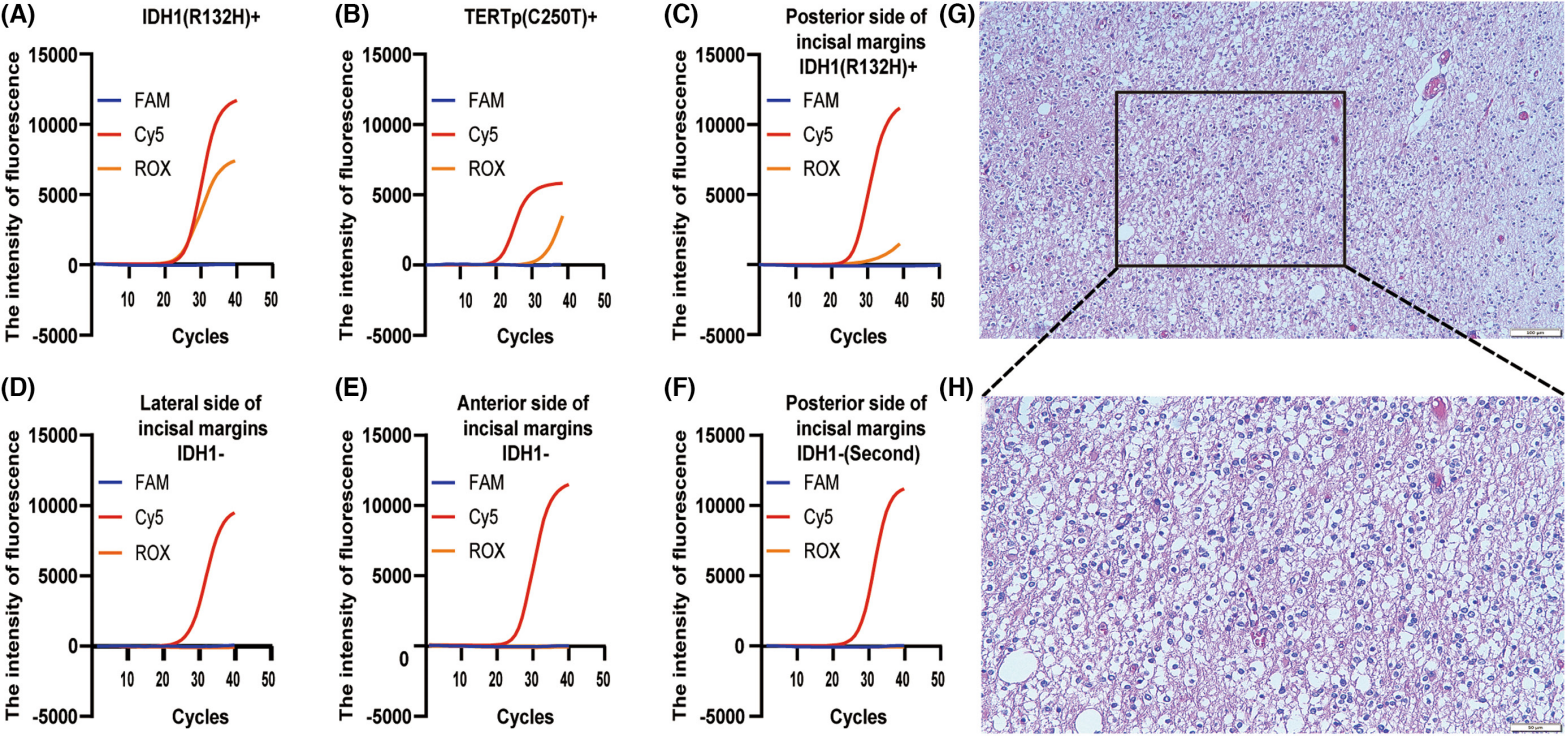
The ROX represents the IDH1 R132H mutation, the FAM represents the IDH1 R132C/G/S mutations, and the Cy5 represents the GAPDH internal reference in the IDH1 real‐time fluorescence curve. The ROX represents the TERTp C250T mutation, the FAM represents the TERTp C228T mutation, and the Cy5 represents the GAPDH internal reference in the TERTp real‐time fluorescence curve. IDH1 R132H mutation (A) and TERTp C250T mutation (B) were discovered during the first intraoperative rapid molecular diagnosis (Cy5 and ROX S‐shaped curves). In posterior‐side (C) tissues, the IDH1 R132H mutation was still present (Cy5 and ROX S‐shaped curve). There is no IDH1 mutation in tissues selected from the lateral (D) and anterior (E) sides after the first resection (only a Cy5 S‐shaped curve). After further resection, the IDH1 R132H mutation disappeared (F) (only a Cy5 S‐shaped curve). The H&E staining pictures of pathological tissues are 100× (G) and 200× (H), suggesting oligodendroglioma, WHO grade 2.

The success of oncological surgery is increasingly defined by the extent of resection, with the goal of complete removal or maximum safe cell reduction. Numerous studies have shown that maximum safe resection has significant prognostic benefits through improved overall survival (OS) and has become the basis for multidisciplinary treatment of lower and higher‐grade brain tumors. Although multiple surgical guidance and imaging modalities exist, iMRI provides the highest evaluation of image quality for surgical implementation.^9^ Yahanda and colleagues reported that they performed further resections in nearly half (49.7%) of lower‐grade glioma cases due to the application of iMRI.^10^ The intraoperative MRI in our reported cases also showed partial co‐location of abnormal signals with the preoperative MRI lesion, suggesting the possible presence of some tumor remnants.

An increasing number of molecular markers have been proven to play an essential role in the classification, typing, grading, prognosis, and treatment of gliomas.^6^ Therefore, it is significant for surgeons to determine the nature and boundaries of tumors at the molecular level intraoperatively. Currently, there are two main technologies for the rapid detection of gene mutations: mass spectrometry (MS) and PCR. Although MS has the advantage of being fast, it cannot be used for IDH typing or the detection of other molecular mutations. In contrast, AIGS real‐time fluorescence PCR‐based rapid molecular diagnosis can be designed with different primers and probes to detect various molecular mutations (e.g., TERTp), in addition to IDH1.^8^ In addition, we use a highly automated integration of sample detection and result output to compress the overall testing time to 55 min. In this case report, we detected IDH1 and TERTp mutations using AIGS, which both provided the pathologist with mutational information on molecular markers for intraoperative frozen histopathological diagnosis and also validated the accuracy of iMRI in localizing the tumor boundary, providing greater clarity on the location and extent of residual lesions, thus avoiding misinterpretation due to interference of MRI by uncontrollable factors and enabling precise resection of the tumor at the imaging and molecular levels. The patient recovered well after the operation, with no significant functional neurological abnormalities at the last follow‐up.

## CONFLICT OF INTEREST STATEMENT

The authors have no personal, financial, or institutional interest in any of the drugs, materials, or devices described in this article.

## Supporting information


Appendix S1

Video S1

## Data Availability

Research data are not shared.
