# Supplementary figures and images for: Intraoperative rapid molecular diagnosis validates MRI‐based glioma boundary evaluation: A case report
Source: CNS Neurosci Ther. 2023 Apr 23;29(7):2036–9. doi: 10.1111/cns.14233 (PMC10324348; doi:10.1111/cns.14233)

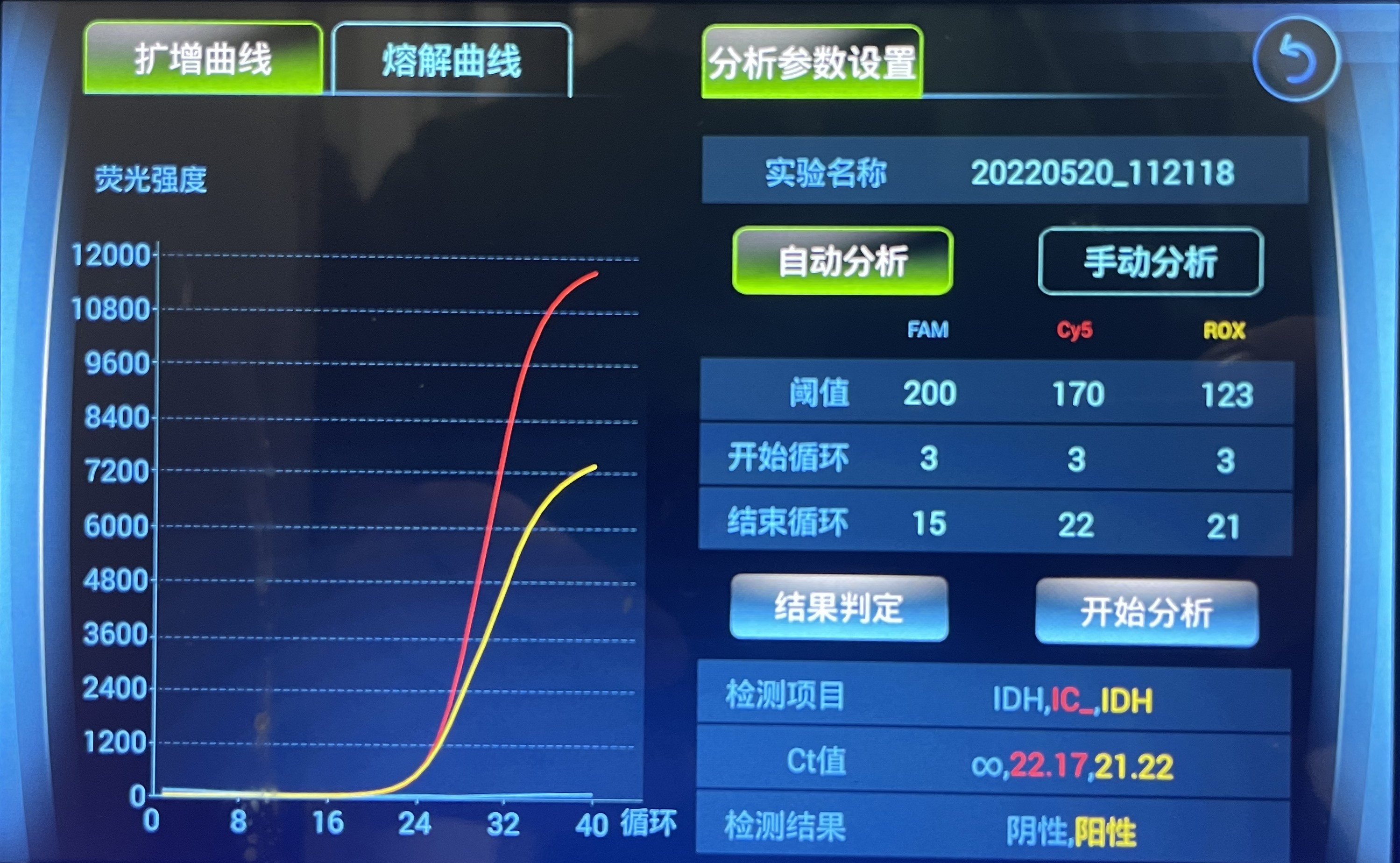

Supplement: Supplementary file 1 — Appendix S1 [file CNS-29-2036-s002.zip › AIGS_FILES/Real time images/IDH1 (Fig2A).jpg]

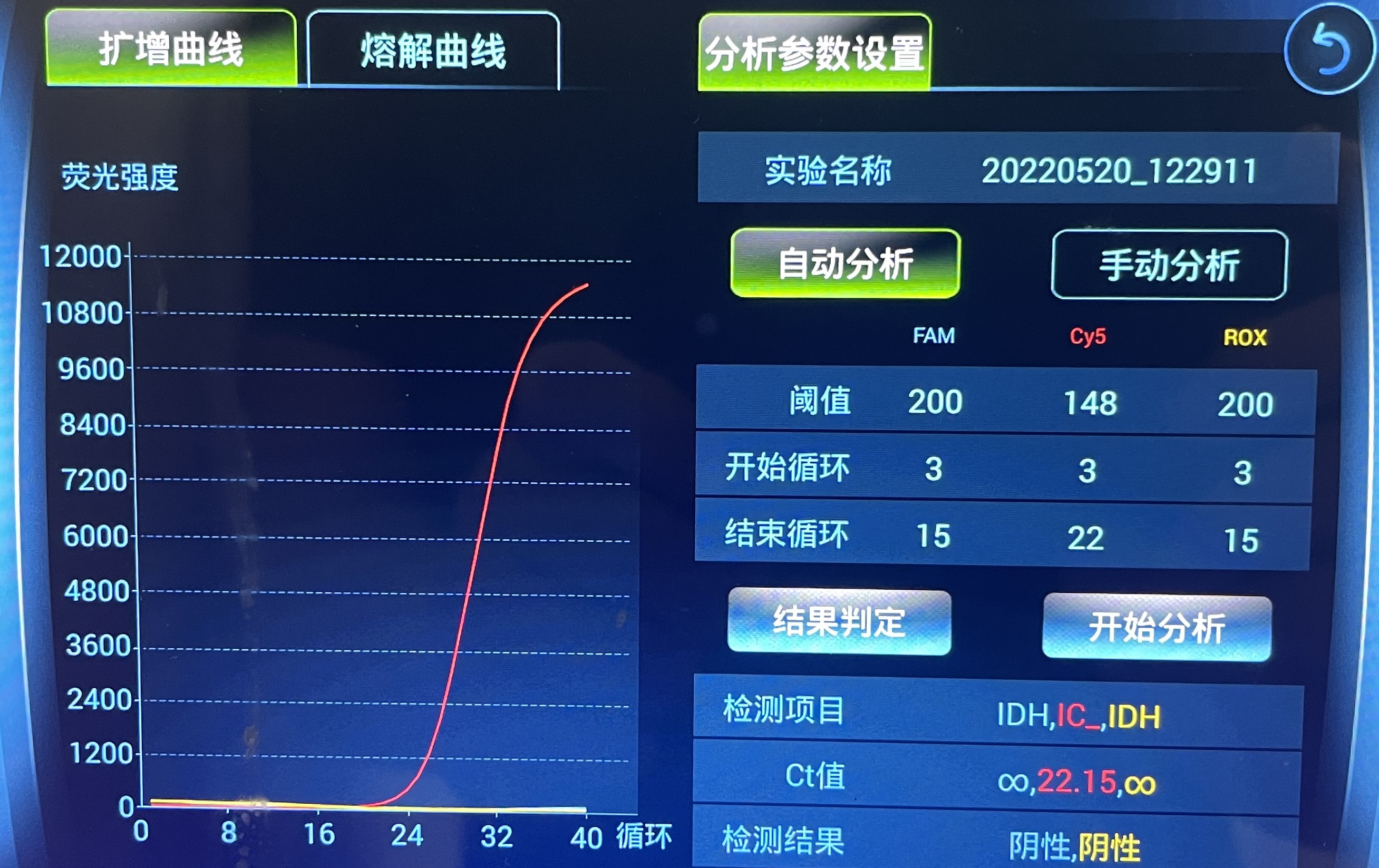

Supplement: Supplementary file 1 — Appendix S1 [file CNS-29-2036-s002.zip › AIGS_FILES/Real time images/IDH1_Anterior side of incisal margins (Fig2E).jpg]

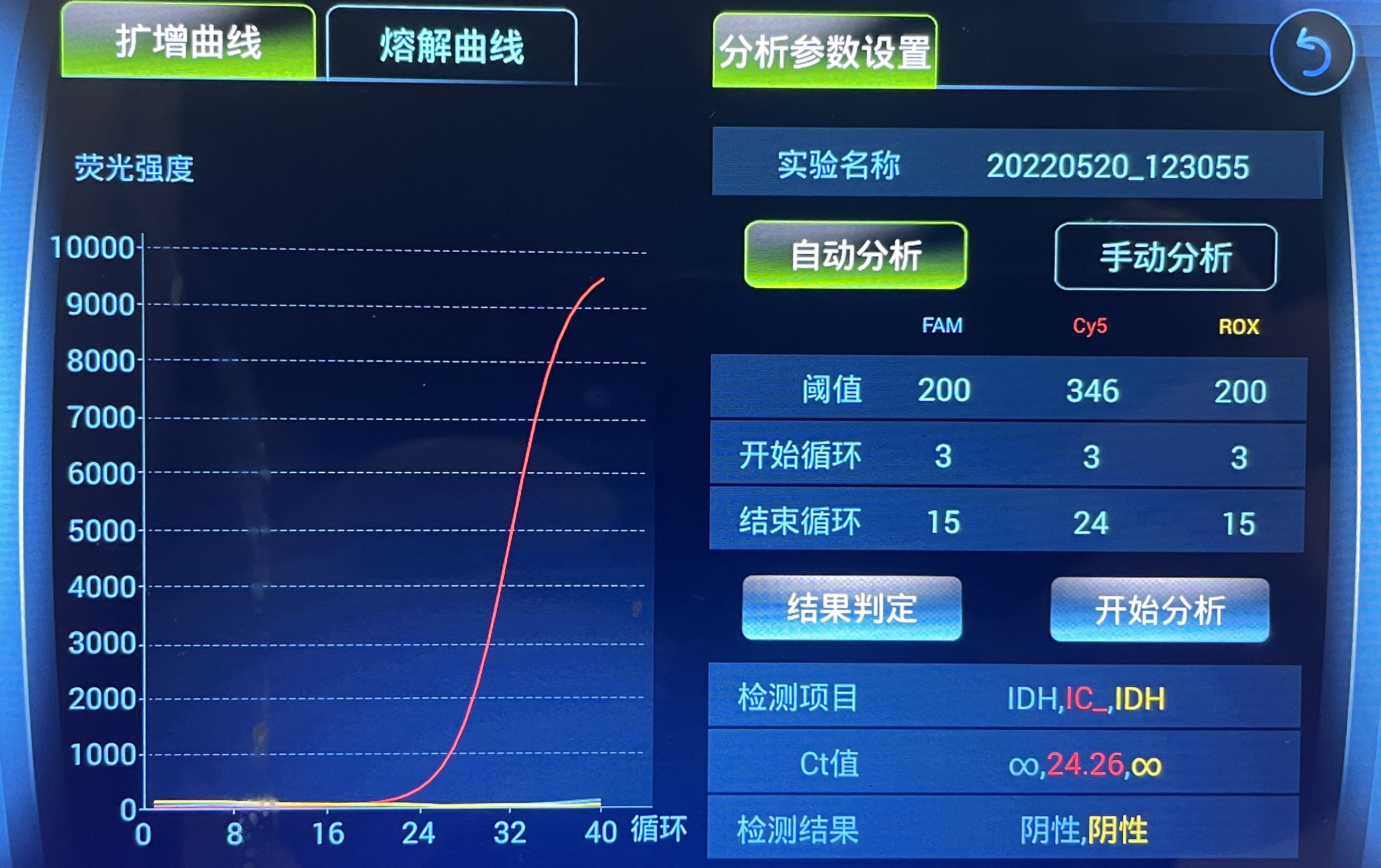

Supplement: Supplementary file 1 — Appendix S1 [file CNS-29-2036-s002.zip › AIGS_FILES/Real time images/IDH1_Lateral side of incisal margins (Fig2D).jpg]

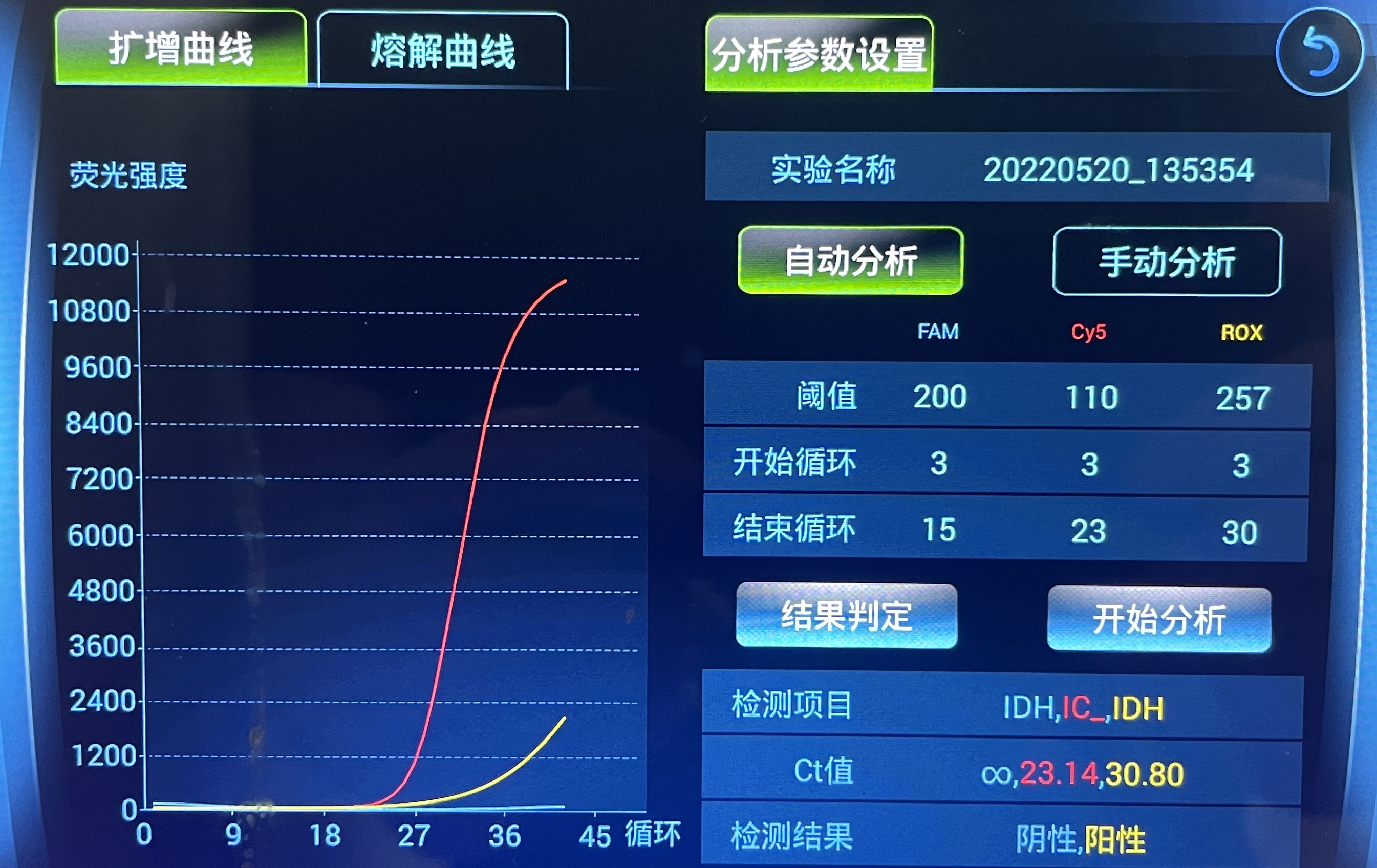

Supplement: Supplementary file 1 — Appendix S1 [file CNS-29-2036-s002.zip › AIGS_FILES/Real time images/IDH1_Posterior side of incisal margins (Fig2C).jpg]

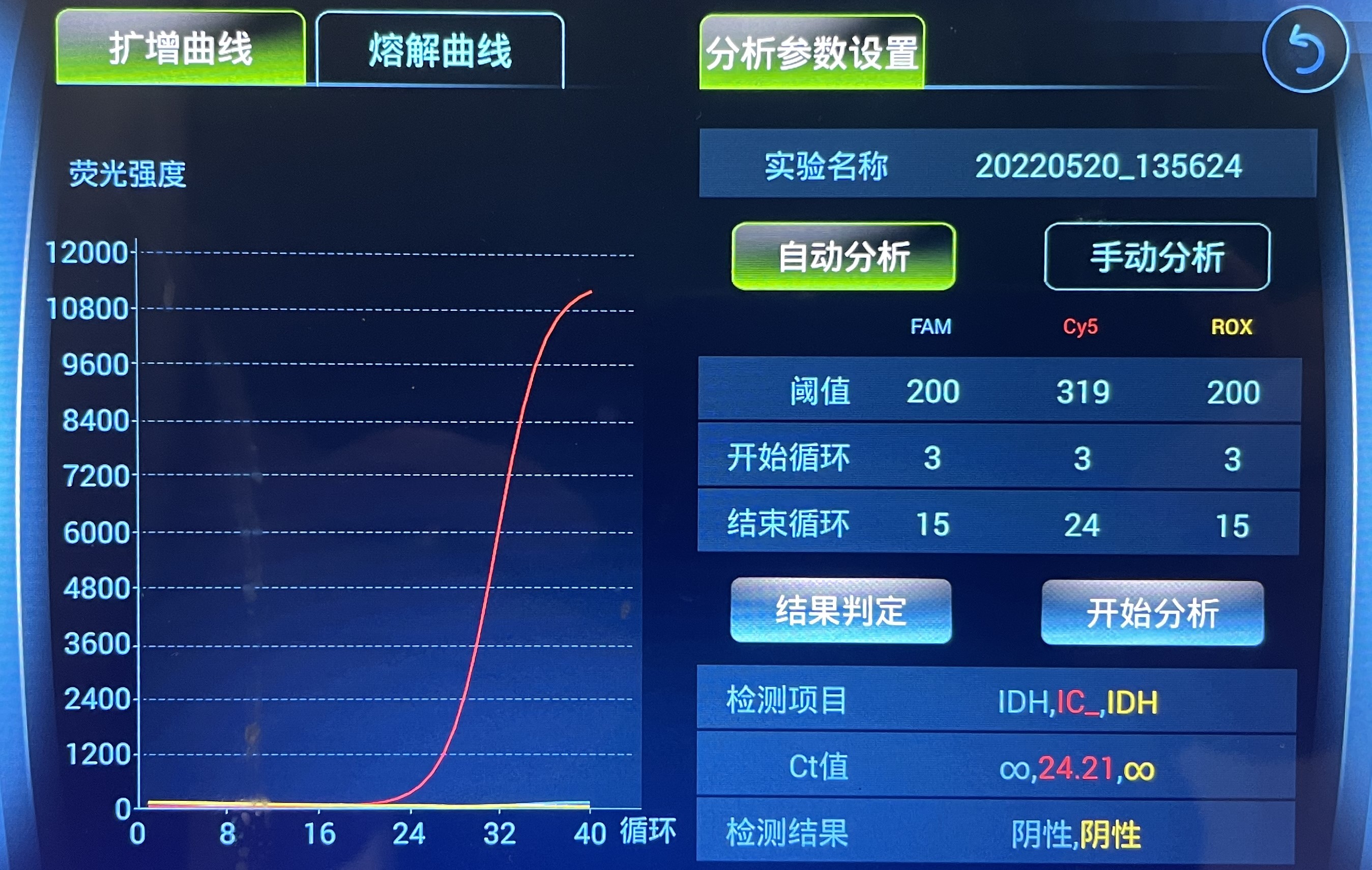

Supplement: Supplementary file 1 — Appendix S1 [file CNS-29-2036-s002.zip › AIGS_FILES/Real time images/IDH1_Posterior side of incisal margins (Second) (Fig2F).jpg]

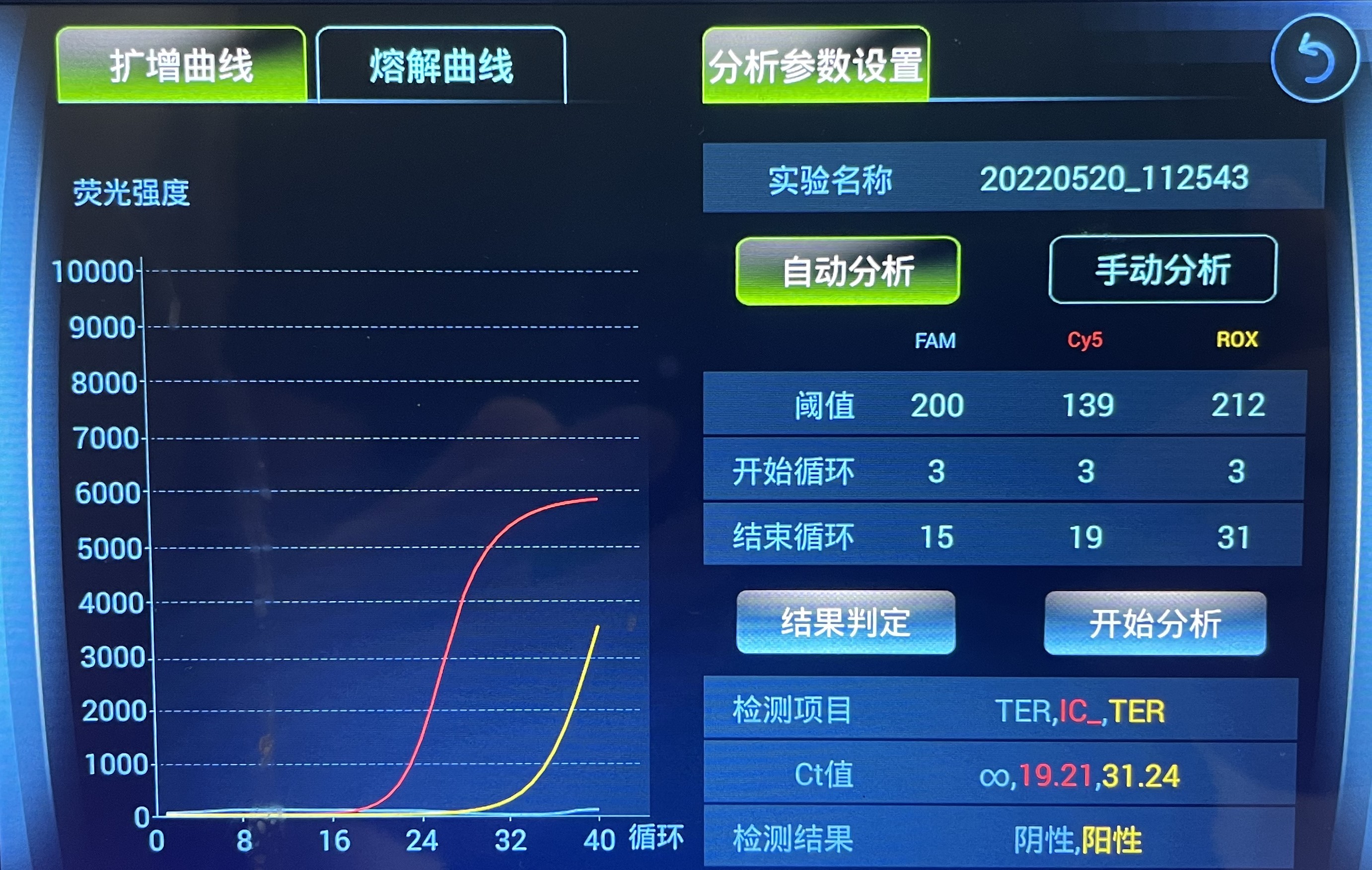

Supplement: Supplementary file 1 — Appendix S1 [file CNS-29-2036-s002.zip › AIGS_FILES/Real time images/TERTp (Fig2B).jpg]
